# Detection of Volatile Organic Compounds by Using MEMS Sensors

**DOI:** 10.3390/s22114102

**Published:** 2022-05-28

**Authors:** Mohamed Arabi, Majed Alghamdi, Khalid Kabel, Ahmed Labena, Walaa S. Gado, Bhoomi Mavani, Alison J. Scott, Alexander Penlidis, Mustafa Yavuz, Eihab Abdel-Rahman

**Affiliations:** 1 Department of Systems Design Engineering, University of Waterloo, Waterloo, ON N2L 3G1, Canada; eihab@uwaterloo.ca; 2 National Center for Electronics and Defense Systems, King Abdulazaiz City for Science and Technology (KACST), Riyadh 12354, Saudi Arabia; 3 Department of Petroleum Applications, Egyptian Petroleum Research Institute (EPRI), Cairo 11727, Egypt; drkhalid1977@yahoo.com; 4 Department of Processes Design & Development, Egyptian Petroleum Research Institute (EPRI), Cairo 11727, Egypt; labena.labena@gmail.com; 5 Department of Petrochemicals, Egyptian Petroleum Research Institute (EPRI), Cairo 11727, Egypt; walaa_shabaan86@yahoo.com; 6 Department of Chemical Engineering, Institute for Polymer Research (IPR), University of Waterloo, Waterloo, ON N2L 3G1, Canada; bmavani@uwaterloo.ca (B.M.); ajscott@uwaterloo.ca (A.J.S.); penlidis@uwaterloo.ca (A.P.); 7 Department of Mechanical and Mechatronics Engineering, University of Waterloo, Waterloo, ON N2L 3G1, Canada; myavuz@uwaterloo.ca

**Keywords:** electrostatic, bifurcation, MEMS, VOCs

## Abstract

We report on the deployment of MEMS static bifurcation (DC) sensors for the detection of volatile organic compounds (VOCs): hydrogen sulfide and formaldehyde. We demonstrate a sensor that can detect as low as a few ppm of hydrogen sulfide. We also demonstrate a sensor array that can selectively detect formaldehyde in the presence of benzene, a closely related interferent. Toward that end, we investigate the sensitivity and selectivity of two detector polymers—polyaniline (PANI) and poly (2,5-dimethyl aniline) (P25DMA)—to both gases. A semiautomatic method is developed to functionalize individual sensors and sensor arrays with the detector polymers. We found that the sensor array can selectively sense 1 ppm of formaldehyde in the presence of benzene.

## 1. Introduction

Volatile organic compounds (VOCs), such as hydrogen sulfide (H_2_S), formaldehyde (HCHO), and benzene (C_6_H_6_) are widely released into ambient air as a result of industrial processes. Various forms of hydrogen sulfide produced by naturally occurring sulfate-reducing bacteria pose serious industrial process challenges to oil pipeline operators [1]. Formaldehyde vapor in indoor environment is a known health hazard [2]. Furniture and paint are major sources of formaldehyde vapor indoors [2]. Frequent exposure to hydrogen sulfide can be lethal and infrequent exposure can lead to serious ocular, respiratory, neurological, cardiovascular, metabolic, and reproductive effects [3,4].

Gas sensors can monitor the presence and concentration of VOCs in ambient air and provide a tool to protect against these dangers. The most common technologies used for gas sensing are solid-state sensors, electrochemical sensors, catalytic sensors, ionization sensors, and microelectromechanical systems (MEMS) sensors [5]. Small size, high sensitivity [6], and compatibility with smart electronics technology [7] are important advantages for MEMS sensors.

MEMS is a batch-fabricated microscale system that performs actuation or sensing functions. MEMS sensors convert the physical quantity into easily read out electrical signals. The MEMS sensors have many applications such as accelerometers [8], gyroscopes [9], pressure sensors [10], and gas sensors [6] wherein the physical quantities measured are accelerations, angles, pressures, and concentration of gases, respectively. MEMS are transducers as they transfer the energy between at least two domains [11]. The transduction methods in MEMS sensors can be piezoelectric, piezoresistive, and electrostatic methods [12]. Electrostatic transduction is the most popular actuation and sensing method in MEMS [12]. Electrostatic transduction is fast and has low power consumption when compared to electrothermal transduction [12]. It doesn’t need an external field source as the electromagnetic transduction [12].

Inertial MEMS sensors are functionalized with detector material to absorb a target gas. They detect gas concentration via the resulting change in mass. The most widely used detector materials are polymers [6] and metal oxides [13,14]. Recently, Mistry et al. have built a MEMS sensors from the detector metal oxide itself rather than adding it as an extra layer [14].

Conducting polymers, such as polyaniline (PANI) and poly(2,5-dimethyl aniline) (P25DMA) are used as sensing materials due to their affinity to VOCs, the reversibility of their electrical and optical properties, low cost, and fabrication flexibility [15,16]. Conducting emeraldine PANI salt results from the interaction of blue (non-conducting) and green conducting emeraldine base with an acidic medium, such as H_2_S. Mousavi et al. [17] demonstrated a resistive-type H_2_S sensor based on thin-film PANI. The morphology of PANI can be enhanced to improve its sensitivity to H_2_S by controlling synthesis conditions, such as the oxidant agent, monomer concentration, the ratio of the monomer to oxidant, and the reaction time and temperature [18].

Traditionally, inertial MEMS gas sensors [19,20,21] have employed static and dynamic detection modes. In the static mode, gas sorption is related to a structural displacement under the sorbed mass [21]. For example, Schlicke et al. [21] used the displacement of an electrostatically actuated membranes to detect toluene at concentrations higher than 1000 ppm. In the dynamic mode, gas sorption is related to a frequency shift in a resonant peak [19,20,21,22]. Dam et al. [22] used the shift in the resonance frequency of a clamped–clamped microbeam functionalized with polyacrylic acid (PAAc) to detect sub-ppm concentrations of ammonia in air. Park et al. [23] detected 526 ppm of a VOC (toluene) in dry nitrogen by using a frequency shift in the peak response of a capacitive micromachined ultrasonic transducer (CMUT) functionalized with poly(styrene-co-allyl alcohol) (PSAA). The use of sensor arrays equipped with multiple detector materials has been proposed as a method to improve the sensitivity and selectivity of gas detection. Possas–Abreu et al. [24] demonstrated an array of seven sensors equipped with various detector polymers for the detection of thirteen VOCs. Their best sensitivity was detection of 103 ppm of phenyl acetate in dry nitrogen by using a sensor functionalized with polyacetylene.

Bifurcation sensors were recently introduced [6,25] as alternative inertial MEMS gas sensors. They exploit static and dynamic bifurcations in order to enhance the signal-to-noise ratio (SNR). At those bifurcations, an equilibrium position or a periodic orbit experiences a qualitative change in size due to sorbed gas molecules. They have been shown to increase the size of the response signal to stimulus from a small (incremental) change to a larger (qualitative) change. Khater et al. [6] demonstrated a static bifurcation MEMS sensor made of a cantilever beam functionalized with P25DMA. They successfully detected 5 ppm of ethanol vapor in dry nitrogen. Kumar et al. [26] and Al-Ghamdi et al. [25] demonstrated dynamic bifurcation sensors made of microcantilever beams. Functionalized with poly 4-vinyl pyridine, Kumar et al.’s [26] sensor was able to detect methanol vapor in dry nitrogen whereas Al-Ghamdi et al.’s [25] sensor, functionalized with P25DMA, detected 100 ppb of ethanol vapor in dry nitrogen.

In this work, we report on development of static bifurcation sensors to detect hydrogen sulfide as well as to detect formaldehyde and differentiate it from an interferent gas (benzene). We also report on the development and deployment of a novel method to functionalize those gas sensors.

## 2. Sensor Design and Characterization

**Figure 1:**
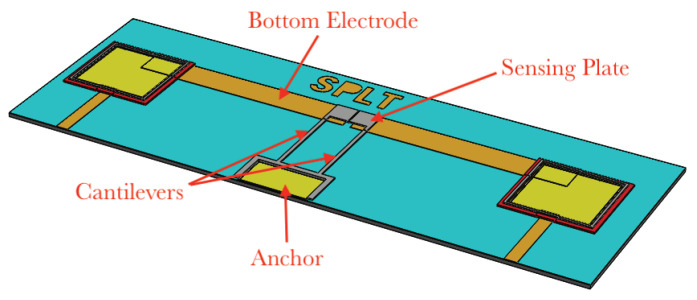
Layout of the gas sensor.

The gas sensors were fabricated by using a surface micromachining process, PolyMUMPS [27]. They are made of a 60 μm×30 μm sense-plate supported by two 125 μm×5 μm microbeams, Figure 1, fabricated in the Poly2 structural layer with a thickness of 1.5 μm. The plate design balances the need for a large surface area to facilitate sensor polymer deposition against the need to eliminate release holes that may lead to leakage of the carrying medium during functionalization. The bottom electrode is patterned underneath the sense-plate in the Poly0 layer.

Modal analysis of the sensor was carried out by using the finite element modeling (FEM) package COMSOL Multiphysics. The 3D model of the sensor was built by defining the layout in the package MEMSPro and simulating PolyMUMPS fabrication steps in it. The resulting 3D model was imported into COMSOL. The mesh was made of 22,309 tetrahedral elements, 13,746 triangular elements, 1724 edge elements, and 100 vertex elements with a total of 125,187 degrees of freedom (DOF). The minimum element size was 1.5 μm. The density, Young’s modulus, and Poisson’s ratio of the structural polysilicon were defined as per the fabrication process handbook [27,28]. The boundary conditions imposed represent fixed support of the beams at the anchor.

**Figure 2:**
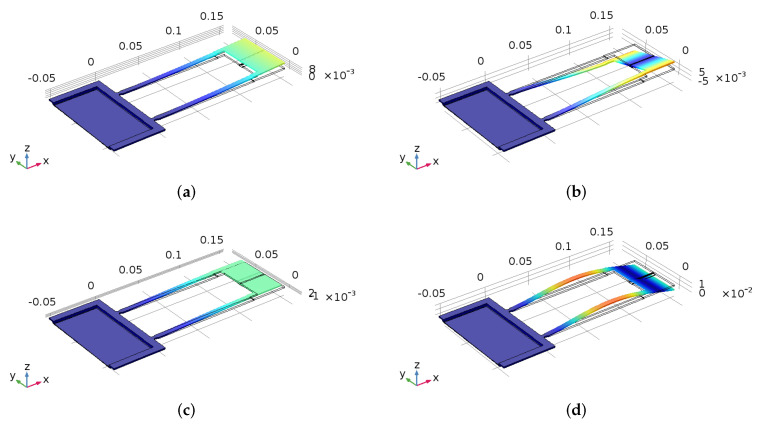
The first four modes of the gas sensor. (**a**) 1st out-of-plane bending; (**b**) 1st torsional mode; (**c**) 1st in-plane bending; (**d**) 2nd out-of-plane bending.

The first four mode shapes of the sensor obtained by applying the eigenfrequency solver to the sensor structure are shown in Figure 2. The fundamental mode is the first out-of-plane bending mode with a natural frequency of $f_{o,1}=36$ kHz. The second mode shape is the first torsional mode (twists around *x*-axis) with $f_{t,1}=209$ kHz. The third mode is the first in-plane bending mode with $f_{i,1}=260$ kHz. The fourth mode shape is the second out-of-plane bending mode with a corresponding natural frequency of $f_{o,2}=385$ kHz. This distribution of the modes over the frequency domain indicates that modal interactions, with their concomitant complications to sensor operation, are unlikely.

**Figure 3:**
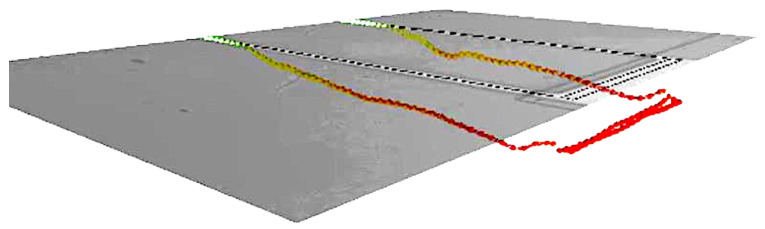
Experimentally obtained first out-of-plane bending mode shape of the sensor.

**Figure 4:**
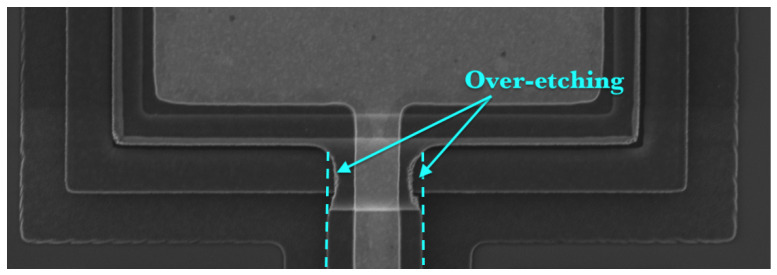
Defects of microfabrication processes.

The response of the fabricated sensor was investigated experimentally under thermal noise excitation by placing it in a vacuum chamber under a pressure of 3 mTorr. A multi-point scan of the top surface of the sensor, Figure 3 was captured by using a laser Doppler vibrometer (LDV). The results show that the dominant mode of response was the first out-of-plane bending mode. The first natural frequency of the sensor was found to be 34 kHz. We suggest that the difference between the measured and calculated natural frequencies is due to the microfabrication artifacts shown in Figure 4. The overetching of the cantilever beam at its fixed end reduces the stiffness of the fabricated sensor.

## 3. Detector Materials

### 3.1. Formaldehyde and Benzene

Two polymeric materials were analyzed for sensitivity and selectivity to formaldehyde and benzene: polyaniline (PANI) and poly(2,5-dimethyl aniline) (P25DMA). The experimental test setup for sorption studies has been described previously in Stewart et al. [15]. Each polymeric sensing material is exposed to a gas, and the amount of analyte that sorbs onto the sensing material is measured. If the sensing material being evaluated is sensitive to the target analyte, higher quantities of the analyte are sorbed. All sorption measurements are taken at room temperature (22 °C) and approximately 15 psi.

The setup exploits a difference in gas concentration (before and after exposure to the sensing material) to establish how much of the target analyte has been sorbed. Before exposure, a “blank” run can be analyzed by a highly accurate Varian 450 gas chromatograph (GC) (with a specialized photon discharge helium ionization detector (PDHID)) to determine the gas concentration for the case of no sorption. After exposure to the sensing material, the gas stream flows into the GC, which can distinguish between similar analytes and record concentrations down to the ppb level.

### 3.2. Hydrogen Sulfide

Emeraldine polyaniline hydrochloride (PANI) was used as a detector of H_2_S. Aniline hydrochloride 0.2 M (2.59 g) was dissolved in 50 mL of distilled water (DW) and 0.25 M (5.71 g) of Ammonium persulfate (APS) was dissolved in 50 mL DW. Both solutions were stored for 1 hour at room temperature then mixed together, stirred, and left to polymerize. After 24 hours, the precipitate was filtered, washed with 300 mL of HCl (0.2 M) and acetone, then dried in a vacuum oven at 60 °C to obtain (PANI) powder.

The chemical structure and functional groups of the prepared PANI were characterized by both a Nicolet iS10 FT-IR spectrophotometer (Thermo Fisher Scientific, Waltham, MA, USA) in the wavenumber range of 500–4000/cm and SENTERRA II Dispersive Raman Microscope (Bruker Optics, Billerica, MA, USA). The PANI molecular weight was recorded by using gel permeation chromatography (GPC), a GPC-Waters 2410 with a refractive index detector using 4 columns styragle HR THF 7.8×300 mm. The H_2_S gas concentrations were measured by an Agilent 7890 Gas Chromatograph (column DB-1—60 m, 60×530 μm×5.0 μm), and GC oven programs are 40 °C (5 min) to 290 °C (5 min) at 250 °C/min.

The GPC analysis confirms the formation of PANI throughout the molecular weight measurement which increases from 93.13 of aniline monomer to 15,080 of PANI. Finally, the morphology of the prepared PANI was confirmed by SEM image which also showed the microporous structure of the prepared polymer.

**Figure 5:**
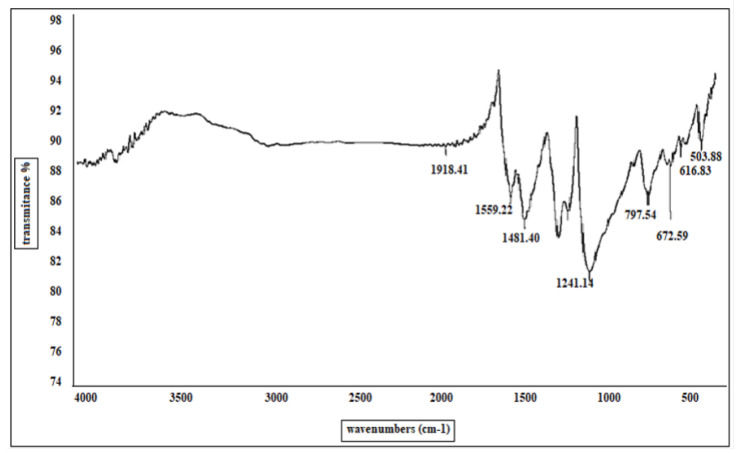
IR spectrum of the prepared PANI.

The FT-IR spectrum (Figure 5) of the prepared powder showed the characteristic peaks of the base form of PANI in the range of 1600–500/cm. The main bands are those located at 1559/cm for C-H bond vibration, 1481/cm for C=C bond vibration, and 1370/cm for C–N bond vibration.

**Figure 6:**
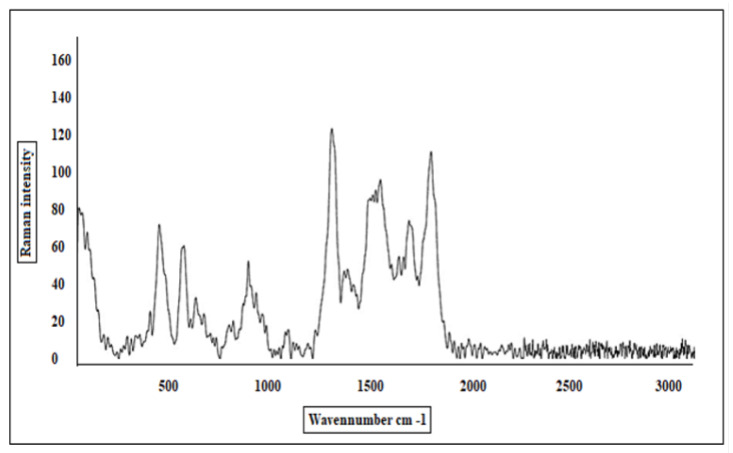
Raman spectrum of the prepared PANI.

**Figure 7:**
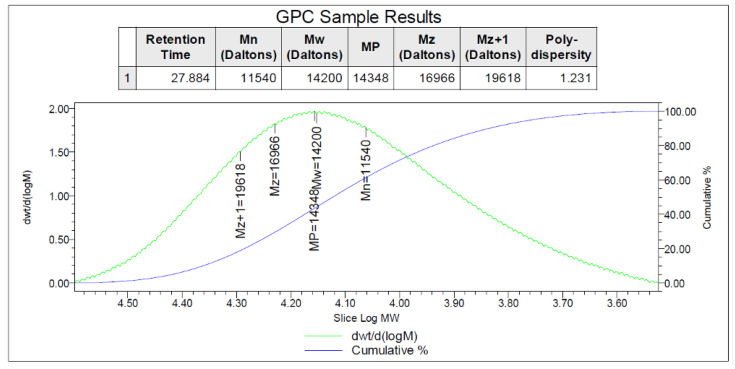
GPC data for PANI.

The Raman spectrum of the prepared PANI (Figure 6) displayed the peak corresponding to the C=C stretching vibrations at 1602/cm with a shoulder at 1636/cm. The C–C vibrations contribute to the band at 1571/cm. The band at 1476/cm corresponds to the C=N stretching vibrations. The sharp bands at 1378/cm and 1414/cm are due to the C N+ ring stretching vibrations. A shoulder observed at 1352/cm is related to the C N+. The molecular weight of the prepared PANI was Mw = 14,200 g/mol and the polydispersity was PDI = 1.3, Figure 7.

**Figure 8:**
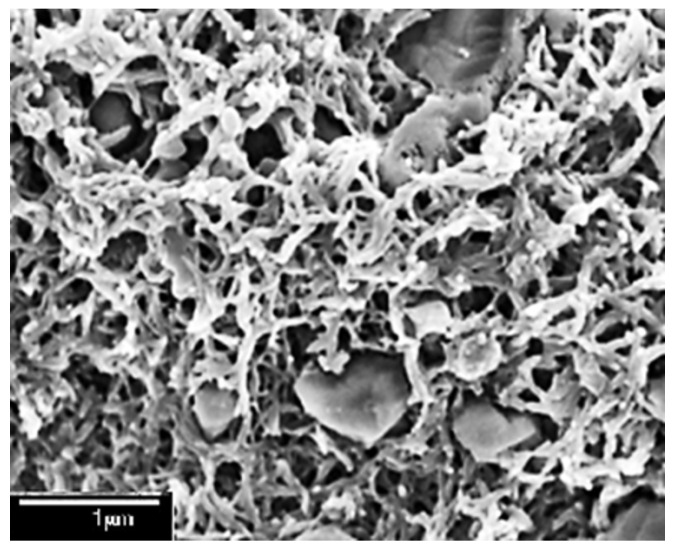
SEM image of the prepared PANI.

The SEM image of the prepared PANI (Figure 8) exhibits the bulky microporous structure of PANI. This type of microstructure increases its sensitivity due to the larger surface area.

## 4. Sensor Functionalization

A semiautomatic deposition method was developed to functionalize gas sensors. One gram of the detector polymer is mixed with fifty grams of a carrying medium. Ethylene glycol C_2_H_6_O_2_ was chosen as a carrying medium due to its low wettability of polysilicon surfaces and fast evaporation rate. The mixture was stirred for 15 min at a speed of 300 RPM to ensure equal dispersion of the detector polymer in the carrying medium.

**Figure 9:**
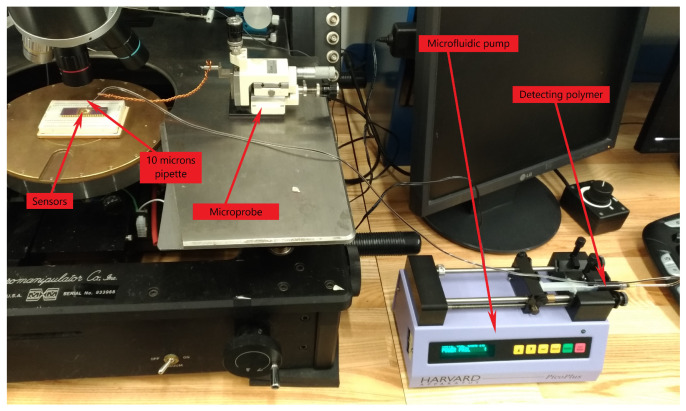
The experimental setup for sensor functionalization.

**Figure 10:**
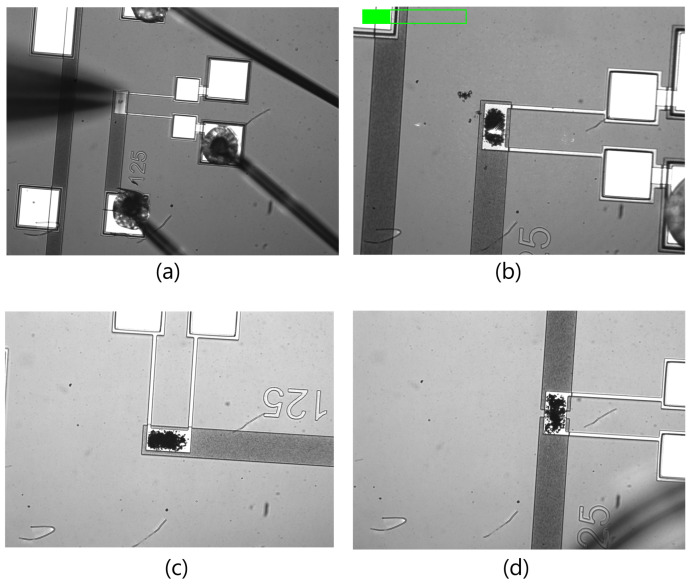
(**a**) The polymer depositing process on the sensor and (**b**–**d**) functionalized sensors.

A microfluidic pump drove the mixture at a flow rate of 0.1 μL/minute through a pipe–pipette assembly supported by a microprobe to deposit a droplet of the mixture on the sense plate. Figure 9 shows the experimental setup. Ethylene glycol was allowed to evaporate naturally leaving the detector polymer on top of the sense plate. The process was continued for at least 10 minutes until the detector polymer was observed to cover most of the sense plate. Figure 10 shows sensor samples during and after functionalization.

**Figure 11:**
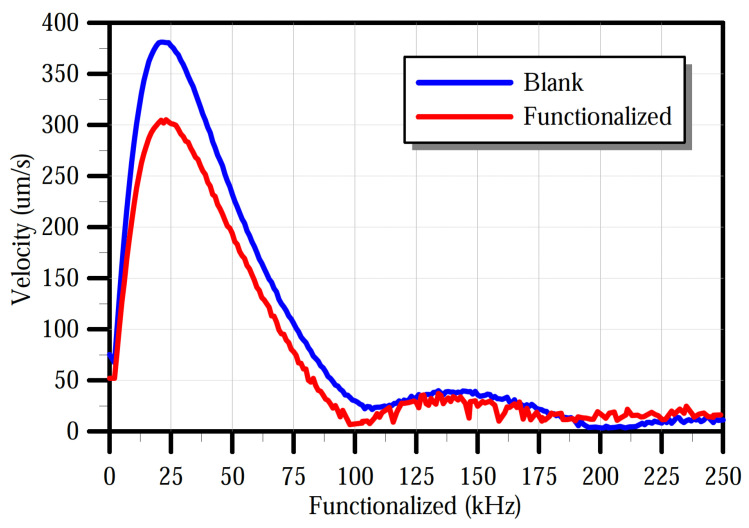
Frequency response of the sensor before and after functionalization with PANI.

The sensors were characterized before and after the functionalization in a dry nitrogen environment inside a gas test chamber. The chamber has a gas inlet, a gas outlet, and four co-axial electric ports that supply the sensor system with three input signals and one digital output channel. The chamber is equipped with a transparent acrylic lid to provide optical access for the LDV. Nitrogen flow was introduced for 15 min. The frequency-response curve of the sensor was obtained by applying a pulse train of 5 V amplitude, 1 kHz frequency, and a 10 μs pulse width. The velocity of the plate’s center point was measured by using the LDV. The fast Fourier transform (FFT) of the measured sensor velocity before and after functionalization was evaluated and plotted in Figure 11. The added mass of the polymer shifted the peak frequency down from 23 kHz to 22 kHz, corresponding to a polymer mass of 0.522 ng, and reduced the peak velocity from 380 mm/s to 295 mm/s.

To measure the static pull-in voltage, a ramp signal varying from 0 V to 6.8 V at a frequency of 5 Hz was applied to the substrate electrode. Pull-in voltage was detected as a sudden change in the plate displacement where the slope of the displacement-voltage curve approaches infinity. The mean and standard deviation of the measured pull-in voltage of five trials were calculated.

The results (Table 1) show that the mean pull-in voltage dropped from 6.543 V to 6.264 V before and after the polymer deposition, respectively. This difference is due to the added mass of the detection polymer. The standard deviation, representing the influence of environmental noise sources, was calculated to be 1.8 mV for the blank and functionalized sensors.

## 5. Experiments

### 5.1. Experimental Setup

**Figure 12:**
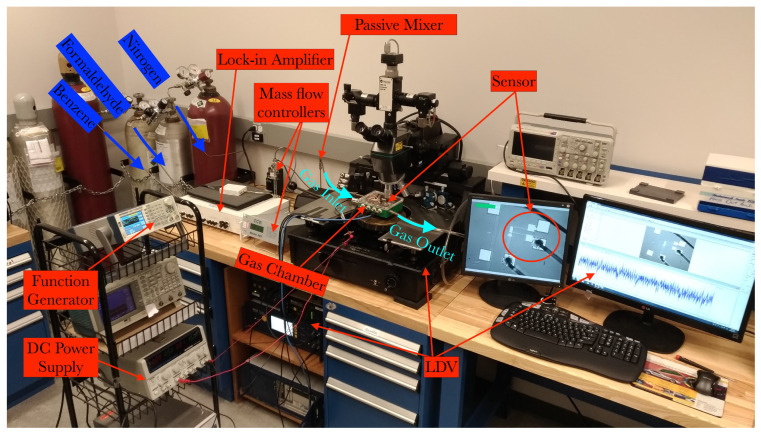
The experimental setup of gas testing.

The sensor die was wire-bonded to a chip carrier, which was placed on a printed circuit board (PCB) inside the gas test chamber. The experimental gas test setup is shown in Figure 12. The sensor was then connected to a function generator to supply DC voltage to the sensor. Figure 12 shows the experimental test setup, consisting of gas tanks connected to a mass flow controller system via a piping system. The gasses are mixed in a passive mixer then supplied into the test chamber where the sensor system is located.

### 5.2. H_2_S Sample Collection and Enrichment

A water sample containing sulfate-reducing bacteria (SRB) was obtained from the General Petroleum Company (GPC), Egypt, with a salinity of 1.6% NaCl. This sample was used as a source of sulfidogenic microorganisms. The sample was inoculated onsite with Postgate’s-B medium and further incubated at 37 °C for 14 days [29,30]. The black precipitate (ferrous sulfide) was used as an indicator for growth and activity. In order to evaluate the hydrogen-sulfide H_2_S production, the SRB sample was further enriched by inoculation with enriched inocula and cultivation at 37 °C for different incubation periods on Postgate’s-C medium [30]. The SRB count was estimated during the cultivation time by using the most probable number (MPN) [31].

The resulting H_2_S gas at different cultivation periods (1, 3, and 5 days) was collected and its concentration was measured by Dräger tubes (2–60 ppm). Furthermore, the measurements were confirmed by gas chromatograph spectrometry (GC). The H_2_S gas concentrations were found to be 20, 40, and 60 ppm respectively.

**Figure 13:**
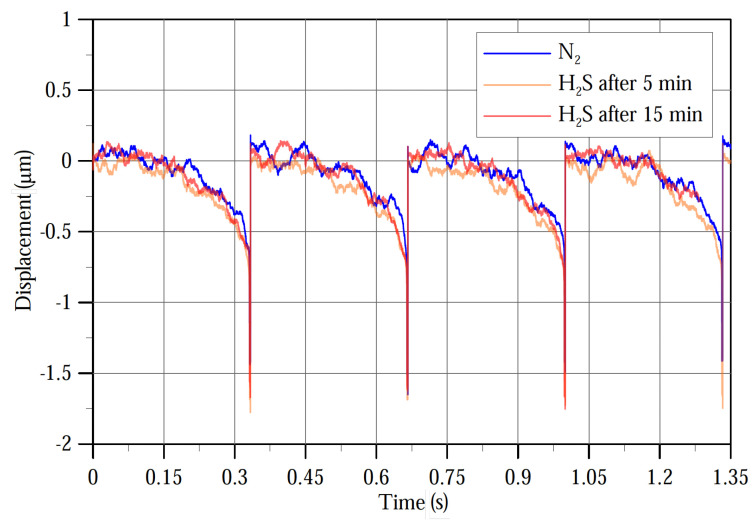
Displacement of the sensor’s tip before and after releasing the gas under a sawtooth signal excitation of 3 Hz frequency.

The target gas was released into the gas chamber by passing a stream of dry nitrogen into the head air of a vial containing an SRB sample cultivated for 1 day. After five minutes of releasing H_2_S into the gas chamber, a sawtooth voltage signal varying from 0 V to 6.4 V at a frequency of 3 Hz was applied to the substrate electrode to detect the pull-in voltage of the sensor. The results plotted in Figure 13 show that the pull-in voltage in the presence of H_2_S was found to be 6.264 V, which is the same value for the pull-in voltage in dry nitrogen, thus indicating failure to detect the gas. The H_2_S release period was then increased to 15 min. The pull-in voltage detected under this condition was 6.257 V. The detection voltage, defined as the difference between pull-in voltage in the presence and absence of H_2_S, was 7 mV proving that the gas can be detected at a 20 ppm concentration under well-mixed conditions.

### 5.3. Formaldehyde/Benzene Detector Sensor

The target analyte gases containing 10 ppm of formaldehyde (F) and 10 ppm of benzene (B) were utilized for evaluations. Pure nitrogen (Praxair grade 5.0) was used to purge samples (and/or parts of the experimental set-up) before actual testing.

Testing the formaldehyde and benzene (F and B) was conducted in two steps. First, we measured each gas individually as a target gas to determine the detection voltage and minimum concentration that can be detected for this gas. Second, two gases were mixed and released in the gas chamber. The formaldehyde was selected as the target gas and the benzene as the interferent gas.

Dry nitrogen gas was introduced into the chamber for fifteen minutes. The dielectric charge on the sensor was measured and recorded. A DC load was then applied to the sensor and increased at a rate of 1 mV/ 30 s until the sensor pulled in. After this, the nitrogen supply was shut down. The pull-in voltage for the nitrogen was calculated and recorded.

The target gas F or B was introduced to the gas chamber at the desired concentration (10 ppm, 5 ppm, 2 ppm, and 1 ppm). The dielectric charge on the sensor and the pull-in voltages were measured. The experiment was repeated at least three times and the results were tabulated. The mean and standard deviation of the detection voltages for both of the detector polymers (PANI and P25DMA) at different levels of target gas concentrations are listed in Table 2.

The results indicate the sensor’s ability to detect both formaldehyde and benzene at low concentrations. PANI was found to be more sensitive to both formaldehyde and benzene than P25DMA. The minimum detectable concentration of formaldehyde using PANI was found to be 1 ppm, whereas the minimum detectable concentration of benzene was found to be 2 ppm for PANI. Therefore, setting the sensor equipped with PANI to a detection voltage of 3 mV allows for the detection of formaldehyde while rejecting benzene. At this level, the detection voltage is well above the circuit noise level at 1.8 mV and the overall sensor repeatability (standard deviation) threshold of 1.8 mV. The minimum detectable concentration of formaldehyde using P25DMA was found to be closer to 5 ppm whereas the minimum detectable concentration of benzene using P25DMA was found to be about 10 ppm.

## 6. Conclusions

We demonstrated the detection of hydrogen sulfide (H_2_S) resulting from SRB by using a static bifurcation MEMS gas sensor. The limit of detection was found to be less than 20 ppm. The sensor was designed and fabricated by using the PolyMUMPS surface micromachining process. The sensor was functionalized with the detector polymer polyaniline (PANI) by using a new deposition method.

Different concentrations of formaldehyde and benzene were tested by using P25DMA and PANI. We found that PANI could detect the formaldehyde down to 1 ppm in dry nitrogen and benzene down to 2 ppm. On the other hand, P25DMA could detect formaldehyde and benzene down to 5 ppm. The sensor could selectively detect formaldehyde in the presence of benzene as an interferent gas.

## Figures and Tables

**Table 1:** Pull-in voltages (V) for the sensor with and without polymer under sawtooth loading.

	Pull-in 1	Pull-in 2	Pull-in 3	Pull-in 4	Pull-in 5	Average	St. Dv
Blank	6.5416	6.5416	6.545	6.5416	6.545	6.54296	0.0018
Functionalized	6.2628	6.2662	6.2628	6.2594	6.26416	6.26212	0.0018

**Table 2:** The mean and standard deviation of the detection voltage, in mV, for combinations of detector polymers and test gases.

	HCHO			
	10 PPM	5 PPM	2 PPM	1 PPM
**P25DMA**	3.6 ± 0.57	1 ± 2		
**PANI**	8 ± 4.73	7.83 ± 8.56	9 ± 8.22	3 ± 1.41
	**C_6_H_6_**			
	**10 PPM**	**5 PPM**	**2 PPM**	**1 PPM**
**P25DMA**	2.66 ± 0.58	−2.33 ± 3.21		
**PANI**	7.5 ± 3.53	7.4 ± 3.64	7 ± 8.2	

## Data Availability

Not applicable.
